# Analysis of Rumen Degradation Characteristics, Attached Microbial Community, and Cellulase Activity Changes of Garlic Skin and *Artemisia argyi* Stalk

**DOI:** 10.3390/ani14010169

**Published:** 2024-01-04

**Authors:** Mingming Gu, Haoyu Liu, Xinghui Jiang, Shuiling Qiu, Keyao Li, Jianing Lu, Mingrui Zhang, Yujun Qiu, Benzhi Wang, Zhiyi Ma, Qianfu Gan

**Affiliations:** 1 College of Animal Science (College of Bee Science), Fujian Agriculture and Forestry University, Fuzhou 350000, China; 13763870270@163.com (M.G.); haoyuliu@163.com (H.L.); jxh0843@163.com (X.J.); shuiling.qiu@aliyun.com (S.Q.); lujn129@163.com (J.L.); zmr15841212112@163.com (M.Z.); q834746958@163.com (Y.Q.); wbz5678@163.com (B.W.); mzy1561566@163.com (Z.M.); 2 Laboratory of Animal Nutritional Physiology and Metabolic Process, Key Laboratory of Agro-Ecological Processes in Subtropical Region, Institute of Subtropical Agriculture, Chinese Academy of Sciences, Changsha 410125, China; 3200609004@fafu.edu.cn

**Keywords:** garlic skin, *Artemisia argyi* stalk, rumen degradation rate, rumen microbiota, cellulase

## Abstract

**Simple Summary:**

Garlic skin and *Artemisia argyi* stalk have broad application prospects as agricultural by-products. However, the current research on them is still relatively limited. Therefore, the focus of this study is to explore the rumen degradation characteristics of these two agricultural by-products, the dynamic changes of surface rumen bacteria and the changes of cellulase activity. Our results showed that garlic skin and *Artemisia argyi* stalk had good rumen degradability and had different effects on rumen bacteria and cellulase activity (β-glucosidase, endo-β-1,4-glucanase, exo-β-1,4-glucanase and neutral xylanase). The results of these studies can better understand the potential of garlic skin and *Artemisia argyi* stalk in the rumen of ruminants and provide a scientific basis for the rational utilization and development of agricultural by-products.

**Abstract:**

The purpose of this study was to study the chemical composition, rumen degradation characteristics, surface attached microbial community and cellulase activity of garlic skin (GS) and *Artemisia argyi* stalk (AS), in order to explain their feeding value. Four 14-month-old healthy Min Dong male goats with permanent rumen fistula were selected as experimental animals. The rumen degradation characteristics of GS and AS were determined by using the nylon bag method, and the bacterial composition, cellulase activity and their relationship on the surface of the two groups were analyzed with high-throughput sequencing of 16S rRNA gene. The results showed that in GS and AS, the effective degradation rate (ED) values of dry matter (DM) were 42.53% and 37.12%, the ED values of crude protein (CP) were 37.19% and 43.38%, the ED values of neutral detergent fiber (NDF) were 36.83% and 36.23%, and the ED values of acid detergent fiber (ADF) were 33.81% and 34.77%. During rumen degradation, the richness and evenness of bacteria attached to the AS surface were higher. At the phylum level, Bacteroidetes and Firmicutes were always the main rumen bacteria in the two groups. At the genus level, fiber-degrading bacteria such as *Prevotella*, *Treponema*, and *Ruminococcus* showed higher levels in GS (*p* < 0.05). Compared with GS, the activity of β-glucosidase (BG enzyme), endo-β-1,4-glucanase (C1 enzyme), exo-β-1,4-glucanase (Cx enzyme) and neutral xylanase (NEX enzyme) attached to AS surface showed a higher trend. Correlation analysis showed that the relative abundance of *Succinivibrio* and *Rikenellaceae_RC9_gut_group* was positively correlated with the rumen degradability of nutrients in GS, and the relative abundance of *Christensenellaceae R-7_group*, *Succinivibrio* and *Ruminococcus* was positively correlated with the rumen degradability of nutrients in AS. The conclusion of this study shows that AS has more potential to become ruminant roughage than GS. In addition, this study also revealed the relationship between cellulase activity and bacteria, which provided new information for us to better analyze the effects of GS and AS on the rumen of ruminants and provided an important theoretical basis for the development and utilization of agricultural by-products.

## 1. Introduction

Due to the continuous rise in animal feed prices and the emergence of food competition between humans and livestock, there is an urgent need to develop new feed resources to alleviate the dependence of the animal feed industry on edible plant resources and improve livestock productivity [1]. Agricultural by-products may cause environmental pollution such as air, soil, and water. However, once reasonably utilized, they will become important resources for promoting sustainable development of animal husbandry and maximizing economic benefits.

Garlic, with a global output of 31 million tons [2], is widely used in herbs and spices around the world. It is rich in polyphenols, such as hydroxybenzoic acid and p-hydroxycinnamic acid, as well as alliin and allicin [3]. These active metabolites play an important role in antioxidant, antibacterial, anti-inflammatory, and anticancer properties [4]. Garlic skin (GS) is the discarded part of garlic after processing, accounting for about 24% of the total weight [5]. It has similar active metabolite components to garlic bulbs. Studies have shown that the total dietary fiber content of GS was 62.10%, which was helpful to promote rumen fermentation, feed intake and crude fiber digestibility during feeding [6,7]. However, we found that there was no previous study on the rumen degradability of GS. *Artemisia argyi* (*A. argyi*), a perennial plant widely distributed in China, is both a medicinal herb and a food source [8]. Studies have shown that *A. argyi* contains a variety of bioactive components such as polysaccharides, flavonoids, and triterpenoids [9]. Its extract has immunomodulatory effects and can effectively fight bacteria, inflammation, and tumors [10]. After extracting specific substances, many stalks or by-products will be produced. Among them, the *A. argyi* stalk (AS) contains bioactive substances and abundant fibers. But most AS are wasted. Currently, we only found research on *A. argyi* as a dietary supplement in Mongolian lambs [11], and there is no research on AS in ruminants.

The rumen of ruminants acts as a natural microbial fermenter, enabling the fermentation of structural carbohydrates such as cellulose and hemicellulose [12]. This fermentation process converts these carbohydrates into volatile fatty acids (VFAs), which serve as a source of energy for the animals. In this process, many microbial communities such as bacteria, fungi and protozoa in the rumen play a major role [13,14]. In the degradation of fiber, rumen bacteria secrete cellulose hydrolases [15], which interact to degrade plant cellulose into easily metabolizable organic compounds [16]. Therefore, a better understanding of the changes in microbial community composition and the cellulase activity during rumen degradation is of great significance for studying the mechanism of feed on rumen fermentation. The purpose of this study was to investigate the effects of GS and AS on rumen degradation characteristics, rumen microbial composition and dynamic changes of cellulase activity. The goal is to provide a theoretical basis for the rational development and utilization of agricultural by-products.

## 2. Materials and Methods

### 2.1. Sample Collection and Chemical Composition

GS and AS were collected in Fuqing, Fujian Province in October. The plant materials are washed with distilled water and then dried in a 65 °C oven for 48 h. After cooling at room temperature for 2 h, the materials are cut into 1–2 cm pieces and pulverized. The resulting powder is sieved through a 40-mesh screen and stored at ambient temperature (22–25 °C) until further use.

Crude protein (CP) and dry matter (DM) content of the roughage were analyzed according to the methods of the Association of Official Analytical Chemists (AOAC) [17]. Neutral detergent fiber (NDF) and acid detergent fiber (ADF) were analyzed with a fiber analyzer (ANKOM 2000i, ANKOM Technology Co., Ltd., New York, NY, USA) following Van Soest et al. [18]. Hemicellulose (HC) was calculated using the equation of HC (%) = NDF (%)–ADF (%). The chemical compositions of GS and AS are shown in Table 1.

### 2.2. Animals Feeding

All experimental procedures involving animals were approved by the Animal Care and Use Committee at the College of Animal Science (College of Bee Science), Fujian Agriculture and Forestry University and followed the recommendations of the European Commission (1997).

Four 14-month-old healthy male Min Dong goats with rumen fistula (body weight 26.60 ± 2.35 kg) were used. The goats were fed twice daily at 09:00 and 17:00; water was always available. The whole experiment was divided into a pre-feeding period of 10 days and a trial period of 49 days. The nutrient content of the forage and concentrate is shown in Table 2.

### 2.3. Ruminal Degradability

The nylon bag method [19,20] was used to analyze the rumen degradation characteristics of DM, CP, NDF and ADF in two samples. The size of the nylon bags was 80 mm × 130 mm, and the aperture was 300 mesh. Then the nylon bags were numbered, dried at 65 °C and weighed. Each sample was carefully weighed to 5 g then placed in a nylon bag. Three parallel nylon bags were set for each sample at different incubation time points of each goat, with a total of 56 nylon bags prepared. Then, 4 h before morning feeding, the “synchronous insertion and batch extraction” method was used to place nylon bags in the rumen and they were removed after incubation for 4, 8, 12, 24, 36, 48, and 72 h. One of the bags was rinsed with running water until the water became clear and then squeezed to remove the water and dried at 65 °C for 48 h to analyze the rumen degradation characteristics. The other bag was rinsed three times with distilled water, squeezed to remove excess water [21], and quickly placed in a −80 °C refrigerator for subsequent bacterial community analysis.

The rumen degradation parameters and effective degradation rates were calculated based on the rumen kinetic index model proposed by Ørskov et al. [22].

P = a + b × (1 − e^−ct^) (1)

ED = a + [b × c/(c + k)](2)

where P is the degradation rate of the sample measured at time t; a is the rapidly degradable fraction (%); b is the slowly degradable fraction (%); c is the constant rate of degradation of b (%/h).; ED is effective degradability (%); and k refers to the fraction rate at which small particles flow out of the rumen. In this study, the value of k was 0.0235%/h [23].

### 2.4. Rumen Microbiome Analysis

The database construction, sequencing, and data analysis parts were completed by OE biotech Co., Ltd. (Shanghai, China). The total genomic DNA of bacteria was extracted according to the instructions of the MagPure Soil DNA LQ Kit (Magen, Shanghai, China). The concentration and purity of DNA were determined by 1.0% agarose gel electrophoresis and a NanoDrop 2000 spectrophotometer (Thermo Fisher Scientific Inc., Wilmington, CA, USA) and stored at −20 °C before use. Amplification of the V3-V4 variable region of the 16S rRNA gene was performed using 343F (5′-TACGGRAGGCAG-3′) and 798R (5′-AGGGGTATCTAATCCT-3′) universal primers. Trimmomatic (v0.36) and FLASH [24] were used for quality control and splicing, respectively. After preprocessing the sequencing data to generate high-quality sequences, using VSEARCH (v2.22.1), the operational classification units (OTUs) were clustered with 97% similarity. QIIME (v1.9.1) was used to select representative sequences of each OTU and compare and annotate all representative sequences with the database. The Silva 16S rRNA database (v.138) was used for comparison and the RDP classifier was used for species comparison annotation, retaining annotation results with a confidence >0.7.

QIIME (v1.9.1) was based on OTUs information alpha and beta diversity analysis. Analyzed using the Chao1, ACE, Shannon, and Simpson Index. The unweighted Unifrac distance matrix was calculated by R (v4.2.3), Principal Component Analysis (PCA) for clustering analysis. Spearman’s rank correlation coefficient was used to test the relationship between variables and the mental test method was used to demonstrate the correlation between major bacterial genera and nutrient degradation. Microbial data analysis and mapping were performed on the OmicStudio cloud platform (https://www.omicstudio.cn/analysis, accessed on 24 October 2023.).

### 2.5. Cellulase Activity Assay

Using a SpectraMax 17 microplate reader (MD, New York, NY, USA) and commercial assay kits (Quanzhou Ruixin Biotechnology Co., Ltd., Fujian, China), the activities of β-glucosidase (BG enzyme), endo-β-1,4-glucanase (C1 enzyme), exo-β-1,4-glucanase (Cx enzyme), and neutral xylanase (NEX enzyme) were measured. Specifically, contents of the nylon bag were weighed separately at 0.1 g and centrifuged at 12,000 rpm for 10 min at 4 °C.

The supernatant was collected to measure the activities of BG enzyme and NEX enzyme. The contents of the nylon bag were separately weighed at 0.2 g and centrifuged at 12,000 rpm for 5 min at 4 °C. The supernatant was discarded, and the precipitate was mixed with pre-chilled 80% ethanol and left to stand at 4 °C for 10 min. After centrifugation at 12,000 rpm for 10 min at 4 °C, the supernatant was discarded, and the precipitate was mixed with pre-chilled extraction buffer and left to stand at 4 °C for 10 min. Subsequently, the mixture was centrifuged at 12,000 rpm for 10 min at 4 °C, and the supernatant was collected to measure the activities of C1 and Cx enzymes.

### 2.6. Statistical Analysis Method

The association between rumen microbiota and dry matter, nutrients, and cellulase activity through Spearman’s correlation analysis. One-way analysis of variance (ANOVA) and Tukey’s test for Data Analysis by SPSS 26.0 (New York, NY, USA). Determine the values of a, b, and c in the Rumen degradation formula through a nonlinear dynamic model by SAS (Cary, NC, USA). Significance was declared at *p* < 0.05.

## 3. Results

### 3.1. Ruminal Degradability

**Figure 1:**
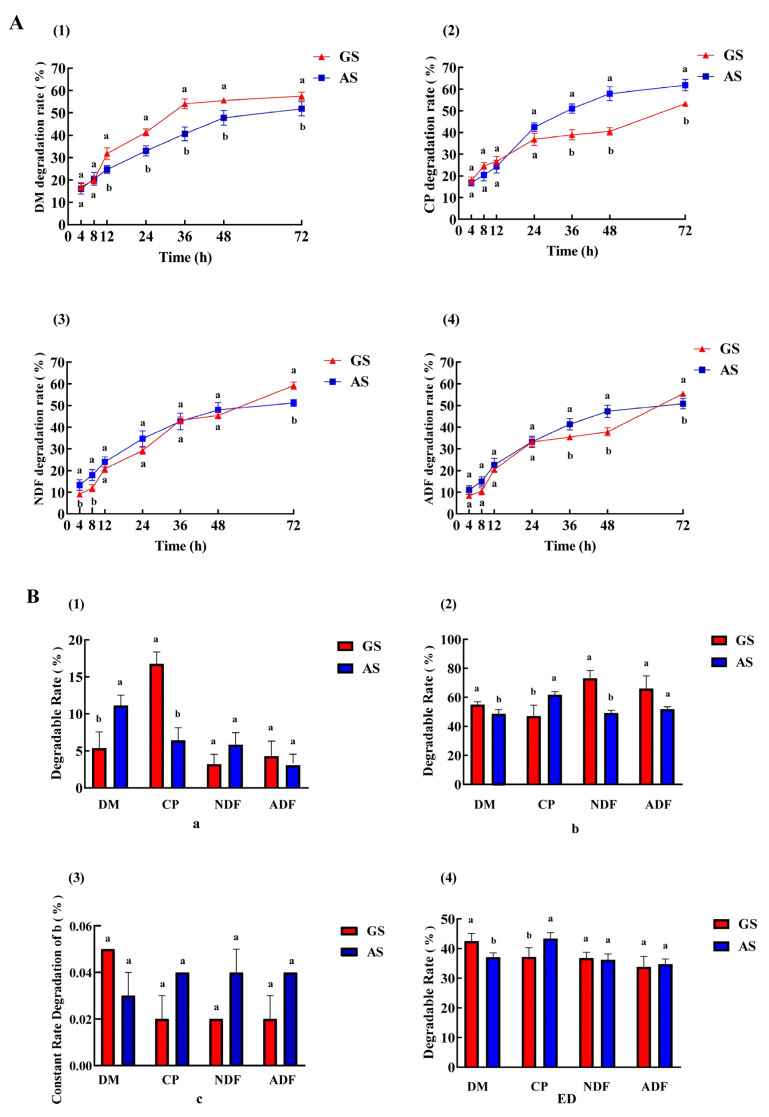
Rumen degradation rate (**A**) and degradation parameters (**B**). GS, garlic skin; AS, *Artemisia argyi* stalk. In (**A**): (1) DM, dry matter; (2) CP, crude protein; (3) NDF, neutral detergent fiber; (4) ADF, acid detergent fiber. Data in the same time points in (**A**) or parameter in (**B**) of the superscript mark different lowercase letters indicate a significant difference (*p* < 0.05). In (**B**): (1) a (%): rapidly de-gradable fraction; (2) b (%): the potentially degradable fraction; (3) c (% / h): the constant rate of degradation of b (% / h); (4) ED (%): effective degradability.

As shown in Figure 1A, the degradation of DM, CP, ADF and NDF in the two groups gradually increased with time. The DM degradation rate of GS at 12 to 72 h was significantly higher than that of AS (*p* < 0.05). The CP degradation of GS at 36 to 72 h was significantly lower than that of AS (*p* < 0.05). The NDF degradation of GS at 4 to 8 h was significantly lower than that of AS (*p* < 0.05), but the degradation rate at 72 h was 59.18%, which was significantly higher than that of AS (51.21%) (*p* < 0.05). The ADF degradation of GS at 36 to 48 h was significantly lower than that of AS (*p* < 0.05), but the degradation rate at 72 h was 55.45%, which was significantly higher than that of AS (50.84%) (*p* < 0.05). In addition, the rumen degradation parameters of the two groups were shown in Figure 1B. In GS, the a of DM was lower than that of AS (*p* < 0.05), and the a of CP was significantly higher than that of AS (*p* < 0.05). The b of DM in GS was significantly higher than that in AS (*p* < 0.05) and the b of CP in GS was significantly lower than that in AS (*p* < 0.05). Additionally, the b of NDF and ADF in GS was significantly higher than that of AS (*p* < 0.05). There was no significant difference in c between the two groups (*p* > 0.05). In GS, the ED of DM was higher than that of AS (*p* < 0.05), and the ED of CP was lower than AS (*p* > 0.05).

### 3.2. Rumen Microbiome Analysis

**Figure 2:**
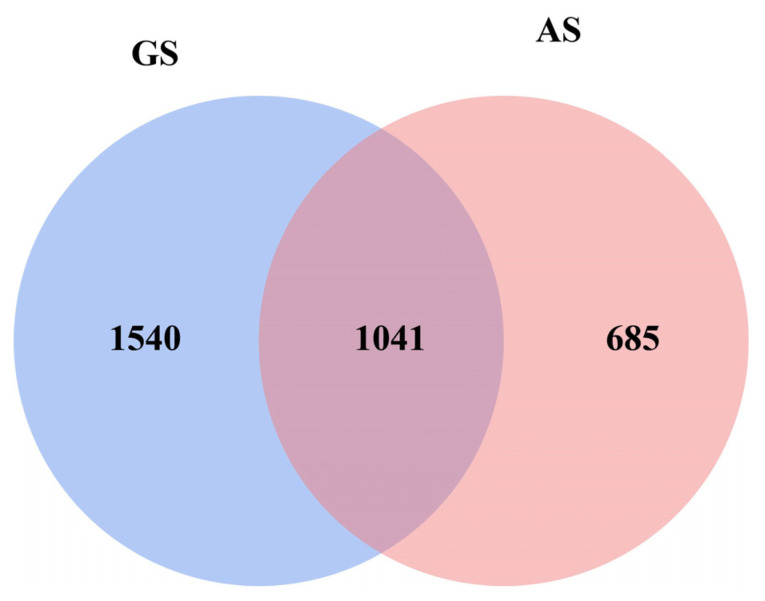
OTU Venn diagram analysis. GS, garlic skin; AS, *Artemisia argyi* stalk.

Through sequencing of rumen bacteria attached to the surface of GS and AS, a total of 3,008,978 raw reads were obtained. Following quality control and filtration, a total of 2,876,618 clean tags were generated, including 1,365,218 clean tags from GS and 1,511,400 clean tags from AS. The dynamic changes of alpha diversity of bacterial communities in the two groups were shown in Table 3 Coverage indicates that the amount of sequencing data is sufficient to reflect the species diversity in the sample. The Chao1 and ACE indices were significantly higher in AS than GS at 12 to 72 h (*p* < 0.05), and the Shannon indices were significantly higher in AS than GS at 72 h (*p* < 0.05). The Venn diagram identified 1041 shared genera between the two groups, with 685 and 1054 unique genera in GS and AS, respectively (Figure 2).

**Figure 3:**
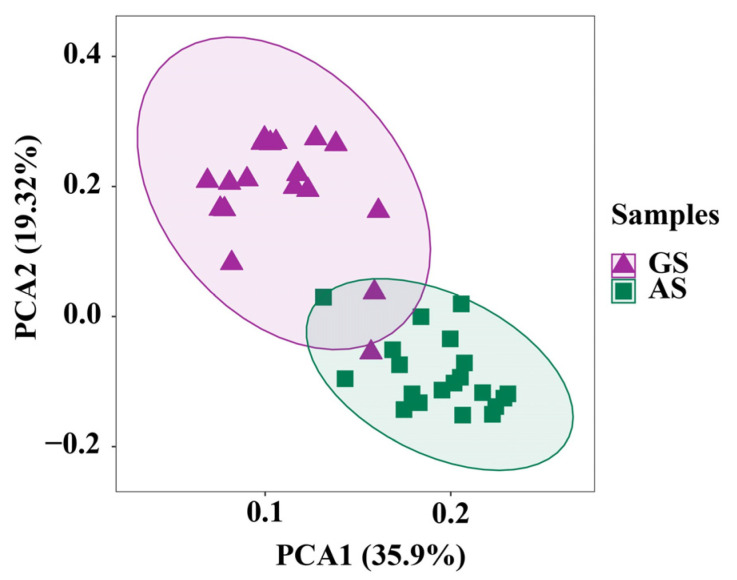
Principal component analysis of rumen bacterial communities attached to GS and AS surfaces. GS, garlic skin; AS, *Artemisia argyi* stalk.

Principal component analysis showed that parts of the two groups were separated from each other, with principal components PC1 and PC2 explaining 35.90% and 19.32% of the variation, respectively (Figure 3).

In this study, a total of 19 bacterial phyla were identified from the microbial communities attached to GS and AS. The four main phyla detected were Bacteroidetes, Firmicutes, Spirochaetes, and Proteobacteria (Table 4).

There was no significant difference in the relative abundance of Bacteroidetes between different time points in GS (*p* > 0.05). In AS, the relative abundance of Bacteroidetes was significantly higher at 12 and 36 h than 72 h (*p* < 0.05) and was also significantly higher than in GS at 36 h (*p* < 0.05). There were no significant differences in the relative abundance of Firmicutes between different time points in GS (*p* > 0.05). In AS, the relative abundance of Firmicutes was significantly higher at 72 h than 12 h (*p* < 0.05). Additionally, there was no significant difference in the relative abundance of Firmicutes between GS and AS (*p* > 0.05). The relative abundance of Spirochaetes was significantly lower at 4 h compared to other time points in GS (*p* < 0.05). In AS, there was no statistical significance (*p* > 0.05), but the relative abundance of Spirochaetes was significantly lower than in GS at 24 and 36 h (*p* > 0.05). The relative abundance of Proteobacteria was significantly higher at 4 h compared to other time points in GS (*p* < 0.05). There were no significant differences among AS groups (*p* > 0.05), but at 72 h, it was significantly higher than in GS (*p* < 0.05).

**Figure 4:**
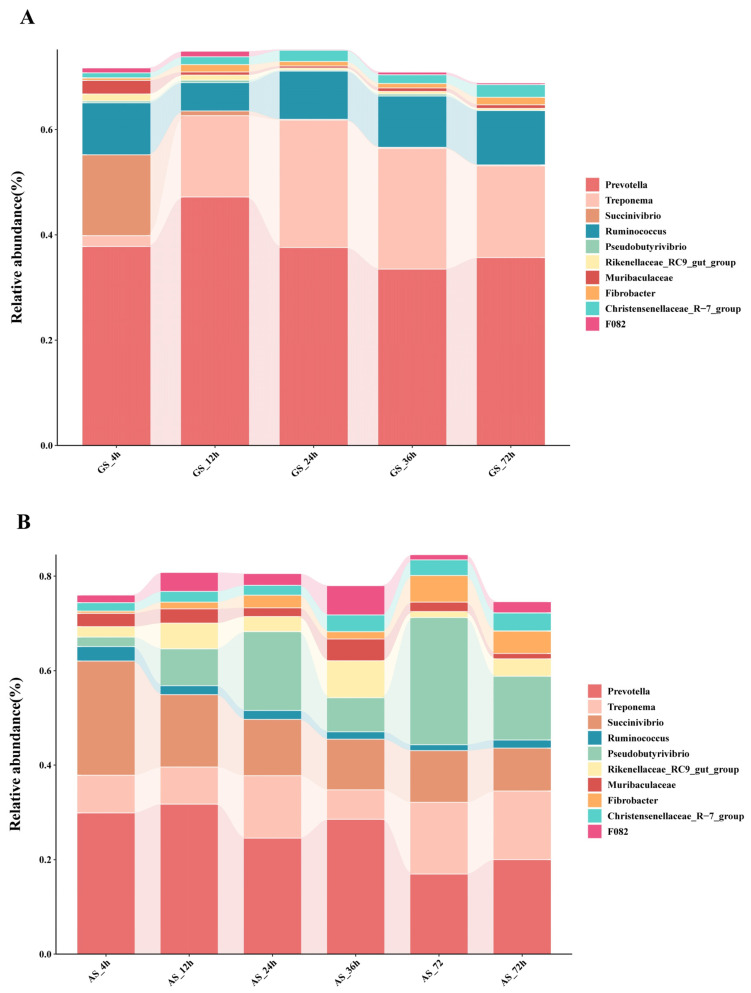
Relative abundance of rumen bacterial genera attached to GS (**A**) and AS (**B**) surfaces.

The top-ten genera are shown in Figure 4. *Prevotella* was the predominant genus of bacteria at each time point in GS and AS. There was no significant change in the relative abundance of *Prevotella* in GS (*p* > 0.05) but was significantly higher in AS at 12 h than at 72 h (*p* < 0.05), and GS was significantly higher than AS from 12 to 36 h (*p* < 0.05). The relative abundance of *Treponema* was significantly lower at 4 h in GS compared to other time points (*p* < 0.05), with no significant changes observed at each time point in AS (*p* > 0.05), and GS was significantly higher than AS at 24 and 72 h (*p* < 0.05). The relative abundance of *Ruminococcus* did not show significant differences between GS and AS at each time point (*p* > 0.05), but GS was significantly higher than AS at 72 h (*p* < 0.05). In addition, other bacterial genera also observed dynamic changes during degradation of the two samples.

### 3.3. Cellulase Activity

Table 5 shows the activity of cellulase activity attached to the surface of GS and AS during rumen degradation. In GS, the activity of BG enzyme was the highest at 36 h, while in AS, it peaked at 24 h. At 4 and 12 h, the activity of BG enzyme of BG in AS was significantly higher than that in GS (*p* < 0.05), while at 36 h, the activity of BG enzyme in GS was significantly higher than that in AS (*p* < 0.05). In GS, the activity of C1 enzyme was the highest at 12 h. In AS, the activity of C1 enzyme peaked at 36 h, and was significantly higher than GS at 4 and 36 h (*p* < 0.05), while GS was significantly higher than AS at 12 h (*p* < 0.05). In GS and AS, the activity of C_X_ enzyme was highest at 72 h and 36 h, respectively. At 12 and 36 h, the activity of C_X_ enzyme in AS was significantly higher than that in GS (*p* < 0.05), but at 72 h, the activity of C_X_ enzyme was significantly higher than that in AS (*p* < 0.05). In GS and AS, the activity of NEX enzyme reached the peak at 72 h and 24 h, respectively. At 12 to 36 h, the activity of NEX enzyme in AS was significantly higher than that in GS (*p* < 0.05), while at 72 h, in GS was significantly higher than that in AS (*p* < 0.05).

### 3.4. The Correlation between Nutrients Degradation Rate, Cellulase Activity and Rumen Bacteria

**Figure 5:**
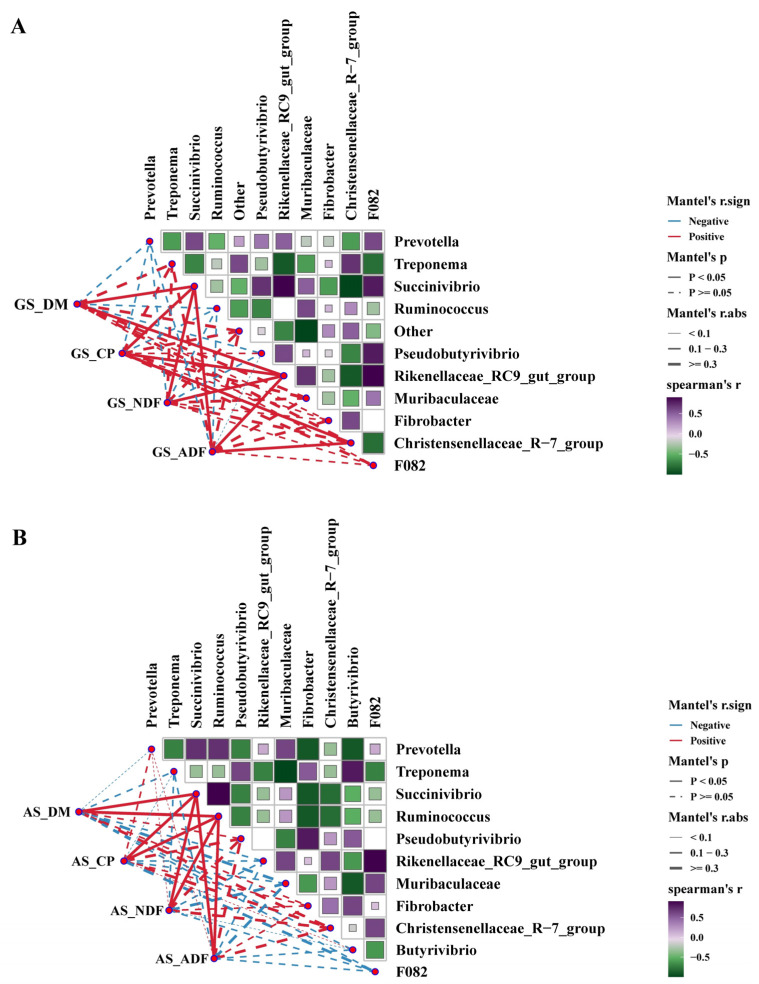
Correlation between nutrient degradation rate of GS (**A**) and AS (**B**) and the relative abundance of the top-ten genera of bacteria. Spearman rank correlation coefficient of >0.5 or <−0.5. Purple indicates positive correlation coefficient. The actual situation of the line represents *p* < 0.05, and the thicker the line, the greater the correlation. The red line represents positive correlation, and the blue line represents negative correlation. Green indicates negative correlation coefficient. GS, garlic skin; AS, *Artemisia argyi* stalk; CP, Crude protein; DM, dry matter; NDF, Neutral detergent fiber, ADF, acid detergent fiber.

The relationship between the relative abundance of the top-ten genera of bacteria and the rumen degradation rate was tested by Spearman’s rank correlation coefficient (Figure 5). In GS, the relative abundance of *Succinivibrio* and *Rikenellaceae_RC9_gut_group* was positively correlated with the degradation rates of DM (*r* = 0.85, *r* = 0.84, *p* < 0.05), CP (r = 0.70, r = 0.82, *p* < 0.05), NDF (r = 0.78, r = 0.76, *p* < 0.05) and ADF (*r* = 0.83, *r* = 0.85, *p* < 0.05). The relative abundance of *Christensenellaceae_R-7_group* was positively correlated with degradation rates of CP (*r* = 0.73, *p* < 0.05) and ADF (*r* = 0.68, *p* < 0.05). In AS, the relative abundance of *Succinivibrio* and *Ruminococcus* was positively correlated with the degradation rates of DM (*r* = 0.97, *r* = 0.94, *p* < 0.05), CP (*r* = 0.85, *r* = 0.68, *p* < 0.05), NDF (*r* = 0.92, *r* = 0.88, *p* < 0.05) and ADF (*r* = 0.98, *r* = 0.91, *p* < 0.05).

**Figure 6:**
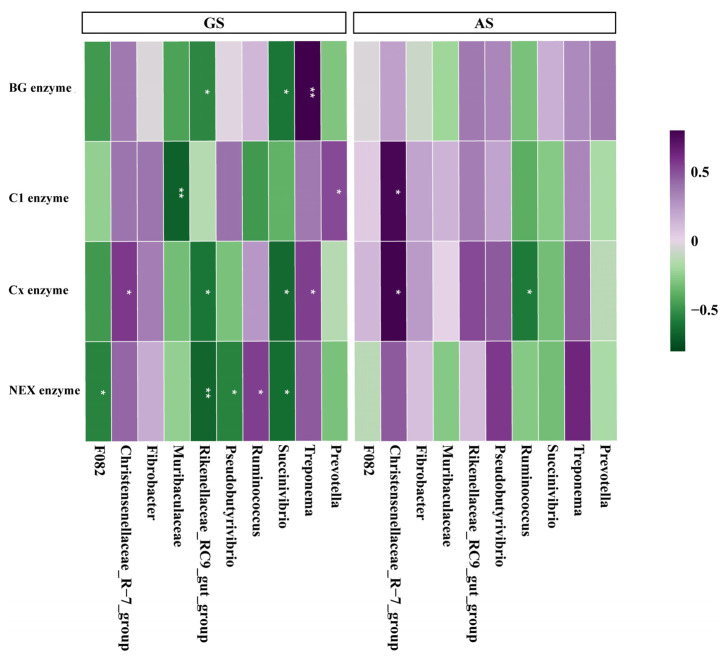
The correlation between the cellulase activity on the surface of GS and AS and the relative abundance of the top ten bacterial genera. GS, garlic skin; AS, *Artemisia argyi* stalk. BG enzyme, β-glucosidase; C1 enzyme, endo-β-1,4-glucanase; Cx enzyme, exo-β-1,4-glucanase; NEX enzyme, neutral xylanase. * *p* < 0.05, ** *p* < 0.01.

The activity of BG enzyme in GS was negatively correlated with the relative abundance of *Succinivibrio* (*r* = −0.60, *p* < 0.05) and *Pseudobutyrivibrio* (*r* = −0.54, *p* < 0.05), and positively correlated with the relative abundance of *Treponema* (*r* = 0.82, *p* < 0.01). The activity of C1 enzyme in GS was negatively correlated with the relative abundance of *Muribaculaceae* (*r* = −0.68, *p* < 0.01), and positively correlated with the relative abundance of *Prevotella* (*r* = 0.52, *p* < 0.05). In AS, it was positively correlated with the relative abundance of *Christensenellaceae_R-7_group* (*r* = 0.58, *p* < 0.05). The activity of Cx enzyme in GS was positively correlated with the relative abundance of *Treponema* (*r* = 0.55, *p* < 0.05) and *Christensenellaceae_R-7_group* (*r* = 0.57, *p* < 0.05), and negatively correlated with the relative abundance of *Succinivibrio* (*r* = −0.64, *p* < 0.05) and *Rikenellaceae_RC9_gut_group* (*r* = −0.60, *p* < 0.05). In AS, it was negatively correlated with the relative abundance of *Ruminococcus* (*r* = −0.53, *p* < 0.05) and positively correlated with the relative abundance of *Christensenellaceae_R-7_group* (*r* = 0.59, *p* < 0.05). The activity of NEX enzyme in GS was negatively correlated with the relative abundance of *Succinivibrio* (*r* = −0.64, *p* < 0.05), *Pseudobutyrivibrio* (*r* = −0.56, *p* < 0.05), *Rikenellaceae_RC9_gut_group* (*r* = −0.66, *p* < 0.01), and *F082* (*r* = −0.54, *p* < 0.05), and positively correlated with the relative abundance of *Ruminococcus* (*r* = 0.54, *p* < 0.05) (Figure 6).

## 4. Discussion

DM degradation is an important index to measure the dry matter intake (DMI) of ruminants [25], which reflects the overall degradation effect of roughage in the rumen [26]. In this experiment, the effective degradation rates of DM in GS (42.53%) was significantly higher than that of AS (37.12%), and the DM degradation rate of GS at 72 h was 57.51%, which was significantly higher than that of AS (51.85%), indicating that GS was more easily decomposed and utilized by the body during digestion [27], resulting in a higher utilization rate in the rumen. In addition, the DM degradation rate at 72 h and ED of GS and AS were higher than those of maize cob (35.80% and 20.90%), distillers grains (34.35% and 26.10%), spent mushroom substrate (49.49% and 28.15%) and rice straw (33.21% and 20.20%) reported by Li et al. [28]. This indicates that the overall rumen degradation performance of GS and AS is better than that of some unconventional and conventional roughages. CP is one of the important indices to evaluate the quality of roughage. The degradation of CP in the rumen is influenced by various factors, including the structural composition of fiber and protein in roughage [29], as well as the capacity of the host’s rumen microbiota to utilize roughage [30]. Research has shown that feeds with high CP content usually led to higher rumen degradability [31]. However, in this study, we found that CP content of GS was significantly higher than that of AS, but the ED of CP and the degradation rate of 36 to 72 h in AS were significantly higher than those in GS. This may be due to differences in experimental animals and feed ingredients. It is worth noting that the b of CP in AS is significantly higher than that in GS, which means that CP in AS can be released slowly in the rumen for a long time, so that the protein can be continuously supplied to the body. NDF and ADF are the most difficult to digest in feed and play an important role in maintaining the digestion and absorption of ruminants and rumen health [32]. Some studies have pointed out that the degradation rate of NDF mainly depends on the b and c value [33]. The ED of NDF and ADF in GS and AS were similar, but the b value of NDF in GS was 73.15%, which was significantly higher than that of AS (49.22%), and the degradation rate of ADF in GS (55.45%) was also significantly higher than that of AS (50.84%) at 72 h. This indicates that NDF in GS can be degraded slowly and continuously by rumen. These results are consistent with previous studies, which reported a negative correlation between rumen degradation and fiber content [34,35].

The rumen bacterial community plays a crucial role in facilitating the continuous extraction of energy from roughage through microbial-mediated fermentation [36]. In this experiment, it was observed that AS had a more abundant rumen bacterial community compared to GS. Furthermore, beta diversity analysis revealed distinct differences in the bacterial community structure attached to the two groups of surfaces. However, GS and AS did not have a significant effect on the overall complexity of rumen microbiota. Firmicutes and Bacteroidetes are the dominant phyla in the rumen microbial community and play a key role in the degradation of roughage [37,38,39], which is consistent with our research findings. Studies have shown that Firmicutes contain many bacteria involved in the efficient degradation and utilization of cellulose [40,41]. In this experiment, the relative abundance of Firmicutes in AS at 72 h was significantly higher than that at 12 h, which may be related to the higher cellulose content in AS. Spirochaetes and Proteobacteria contain a substantial number of cellulose-degrading bacteria [42,43,44,45]. These bacteria degrade roughage, they use fermentation products such as acetic acid and propionic acid as an energy source and provide energy for the host [46]. In this experiment, it was observed that the relative abundance of Spirochaetes and Proteobacteria significantly changed only in the early stage of GS degradation, while it did not show significant changes in AS. However, throughout the subsequent rumen degradation, the abundance of both groups remained relatively stable, indicating that the degradation process of the two roughages did not disrupt the stability of the rumen bacterial community. By comparing the microbial composition between the two groups of roughage, we believed that the differences in the rumen bacterial community during degradation are closely associated with the type of roughage and the rumen incubation time [47,48,49].

*Prevotella* has the highest abundance in the rumen microbial community of ruminants and is closely related to the digestion and metabolism of cellulose, protein, and pectin [50,51,52,53]. Similar results were observed in this experiment. Nutrients such as dry matter and crude protein that are easily degraded in roughage are mainly utilized by microbiota in the early stage of rumen degradation, and gradually consumed with the increase in degradation time. Therefore, the number of *Prevotella* in GS and AS decreased in different degrees in the later stage of degradation, which was consistent with the experimental results of Vahidi et al. [54]. The relative abundance of *Prevotella* in GS was higher than that in AS, which may be due to the low fiber content in GS, and Prevotella tended to mobilize readily available nutrients (such as soluble sugars and proteins) [34]. *Treponema* and *Ruminococcus* are hydrolytic and acid-producing bacteria [55]. Among them, *Treponema* is the main participant in rumen degradation of cellulose [56], and *Ruminococcus* also plays an important role in fiber degradation [57]. In this experiment, when GS and AS were degraded to the middle and late stages in the rumen, we observed that the relative abundance of *Treponema* and *Ruminococcus* in GS was higher than that in AS. In summary, the fiber composition of GS is more conducive to the growth and reproduction of fiber-degrading bacteria in the rumen.

In the rumen of ruminants, microbiota such as bacteria and fungi are symbiotic with the host [58] and degrade more complex structures by decomposing fibers to produce cellulase, including BG enzyme, C1 enzyme, Cx enzyme and NEX enzyme [59]. In this experiment, we observed that the cellulase activity attached to the surface of AS was often higher than that of GS during rumen degradation, indicating that the composition and structure of AS had a positive effect on rumen fiber decomposition ability. Rumen bacteria are closely related to feed quality and cellulase activity [60]. Therefore, we evaluated whether there is a correlation between the genus of bacteria and nutrients and cellulase activity. Spearman’s correlation analysis indicates a strong positive correlation between the degradation of nutrients in GS by the rumen is related to *Succinivibrio*, *Rikenellaceae_RC9_gut_group*, and *Christenselelaceae_R-7_group*. Similarly, there is a strong positive correlation between the degradation of nutrients in AS that is related to *Succinivibrio* and *Ruminococcus*, indicating that the special impact of GS and AS on rumen bacterial communities is due to their different nutritional components. In addition, this experiment found that *Succinivibrio* plays an important role in the rumen degradation of both GS and AS. Previous studies have shown that *Succinivibrio* is among the predominant fiber-dissolving bacteria in the rumen, and its relative abundance is positively correlated with the feed efficiency of ruminants [61]. This is consistent with the results of this experiment. That is, when the relative abundance of *Succinivibrio* decreases, the rate of nutrient degradation gradually slows down. However, in GS, this decrease may be due to the interaction between allicin and microorganisms in the rumen when garlic skin is degraded in the rumen. Allicin has antibacterial and antifungal activity [62], which may lead to the disturbance of rumen microflora, thus inhibiting the growth of some fiber-degrading bacteria and reducing the efficiency of fiber degradation [63]. However, the current understanding of the effects of allicin on rumen microbiota is limited, and further research is needed to fully understand its mechanism. The activity of hydrolases is related to microbiomes that are partially attached to feed particles [64]. In this study, we found that many significantly different bacterial genera were significantly positively correlated with the cellulase activity in two groups. In fact, rumen digestion is the result of a synergistic effect of the microbiota [65]. Studies have shown that the disorder of rumen bacterial community may lead to more complex rumen bacterial network and down-regulate some digestive cellulase activity [66]. Therefore, we speculate that the lower activity of cellulose hydrolase attached to the surface of GS may be caused by the more complex rumen bacterial network. Although no further in-depth research has been conducted, we observed a significant positive correlation between the relative abundance of microbial populations and cellulase activity. This finding provides some insights into potential host-microbe interactions in the rumen and suggests that changes in microbial populations may serve as a mechanism for rumen adaptation to roughage fiber structure.

## 5. Conclusions

In conclusion, there are significant differences in the types of rumen microorganisms attached to the surface of GS and AS and their attachment time, which affect their rumen degradation rate. In this study, although the effective degradation rates of NDF and ADF of GS and AS were similar, the bacterial diversity and cellulase activity on the surface of AS increased significantly during the degradation process. Therefore, we believe that AS as an unconventional feed has greater application potential in goat feeding programs. In addition, *Succinivibrio* was positively correlated with the degradation of nutrients in both groups, revealing the relationship between cellulase and bacteria in the degradation process of rumen fibers of different structures. The results of this study help to better utilize the application of GS and AS in ruminant feed and provide valuable information for rational development of agricultural by-products.

## Figures and Tables

**Table 1:** Chemical composition of GS and AS (% DM basis).

Items	Nutrient Composition				
	DM	CP	NDF	ADF	HC
GS	87.36 ± 0.83 ^b^	12.40 ± 1.02 ^a^	34.17 ± 1.80 ^b^	29.92 ± 1.93 ^b^	4.25 ± 0.69 ^b^
AS	91.27 ± 1.26 ^a^	8.19 ± 0.50 ^b^	83.62 ± 0.39 ^a^	59.54 ± 1.59 ^a^	24.08 ± 1.38 ^a^

GS: Garlic skin, AS: *Artemisia argyi* stalk, DM: dry matter, CP: crude protein, NDF: neutral detergent fiber, ADF: acid detergent fiber, HC: hemicellulose. ^a,b^ Different letters within the same column differ significantly (*p* < 0.05).

**Table 2:** Composition and nutrient composition of the basal diet (DM basis %).

Items	Content
Ingredients	
Leymus chinensis	55.00
Corn	31.00
Soybean meal	12.20
Salt	0.50
Stone powder	0.10
Calcium bicarbonate	0.20
Premix ^1^	1.00
Total	100
Nutrient composition ^2^	
Crude protein	10.94
Neutral detergent fiber	48.66
Acid detergent fiber	24.83
Ether extract	2.36
Calcium	0.62
Phosphorus	0.35
Net energy for lactation (MJ/kg DM)	11.67

^1^ Premix: (% DM) Fe: 90 mg/kg, Cu: 12.5 mg/kg, Mn: 60 mg/kg, Zn: 90 mg/kg, Se: 0.3 mg/kg, I: 1.0 mg/kg, Co: 0.3 mg, Vitamin A: 15,000 IU/kg, Vitamin D: 5000 IU/kg, Vitamin E: 50 mg/kg. ^2^ The net energy of lactation was calculated value; others were measured value.

**Table 3:** Alpha diversity of rumen bacteria attached to GS and AS surfaces.

Items	Time	GS	AS
Chao1	4 h	304.37 ± 16.80	342.71 ± 35.74
	12 h	366.42 ± 24.18 ^B^	499.70 ± 53.82 ^A^
	24 h	313.86 ± 65.66 ^B^	509.87 ± 14.93 ^A^
	36 h	373.45 ± 38.49 ^B^	623.76 ± 61.37 ^A^
	72 h	357.94 ± 59.91 ^B^	584.29 ± 15.51 ^A^
ACE	4 h	340.04 ± 40.60	305.68 ± 18.87
	12 h	362.45 ± 25.67 ^B^	492.93 ± 51.84 ^A^
	24 h	311.99 ± 63.55 ^B^	528.23 ± 24.60 ^A^
	36 h	411.32 ± 34.96 ^B^	620.92 ± 68.00 ^A^
	72 h	354.32 ± 56.36 ^B^	579.46 ± 17.52 ^A^
Shannon	4 h	5.98 ± 0.39	5.59 ± 0.29
	12 h	6.38 ± 0.11	6.73 ± 0.61
	24 h	6.06 ± 0.17	6.47 ± 0.48
	36 h	6.36 ± 0.29	7.18 ± 0.49
	72 h	6.17 ± 0.25 ^B^	6.98 ± 0.27 ^A^
Simpson	4 h	0.94 ± 0.03	0.94 ± 0.05
	12 h	0.96 ± 0.01	0.97 ± 0.02
	24 h	0.96 ± 0.00	0.96 ± 0.01
	36 h	0.97 ± 0.00	0.98 ± 0.01
	72 h	0.97 ± 0.00	0.97 ± 0.02
Coverage	4 h	0.99	0.99
	12 h	0.99	0.99
	24 h	0.99	0.99
	36 h	0.99	0.99
	72 h	0.99	0.99

Different uppercase letters in the same row indicate significant differences (*p* < 0.05). GS, garlic skin; AS, *Artemisia argyi* stalk.

**Table 4:** Relative abundance of rumen bacterial phylum attached to GS and AS surfaces.

Items	Time	GS	AS
Bacteroidetes	4 h	49.58 ± 5.27	42.90 ± 5.04 ^ab^
	12 h	54.83 ± 3.79	50.91 ± 5.57 ^a^
	24 h	43.99 ± 5.84	38.32 ± 8.43 ^ab^
	36 h	40.97 ± 4.11 ^B^	54.07 ± 3.70 ^Aa^
	72 h	44.48 ± 6.62	31.09 ± 7.31 ^b^
Firmicutes	4 h	31.59 ± 5.92	23.07 ± 6.38 ^ab^
	12 h	26.54 ± 6.81	22.90 ± 4.95 ^b^
	24 h	30.32 ± 9.75	32.32 ± 4.40 ^ab^
	36 h	34.42 ± 7.05	25.30 ± 3.94 ^ab^
	72 h	36.10 ± 3.65	38.04 ± 7.46 ^a^
Spirochaetes	4 h	2.07 ± 2.37 ^b^	7.99 ± 10.73
	12 h	15.41 ± 2.94 ^a^	7.97 ± 4.30
	24 h	24.23 ± 4.59 ^Aa^	13.36 ± 2.80 ^B^
	36 h	22.93 ± 3.00 ^Aa^	6.37 ± 3.63 ^B^
	72 h	17.43 ± 6.46 ^a^	14.91 ± 0.87
Proteobacteria	4 h	16.04 ± 9.48 ^a^	24.95 ± 14.52
	12 h	1.69 ± 0.69 ^b^	16.08 ± 13.49
	24 h	0.53 ± 0.56 ^b^	12.66 ± 9.68
	36 h	0.68 ± 0.22 ^b^	11.56 ± 8.36
	72 h	0.42 ± 0.19 ^Bb^	10.15 ± 5.78 ^A^

Different lowercase letters in the same column indicate significant differences in data (*p* < 0.05), while different uppercase letters in the same row indicate significant differences (*p* < 0.05). GS, garlic skin; AS, *Artemisia argyi* stalk.

**Table 5:** Cellulase activity attached to the surface of GS and AS during rumen degradation.

Items	Time	GS	AS
BG enzyme (nmol/min/g)	4 h	118.01 ± 10.2 ^Bd^	141.97 ± 3.7 ^Ab^
	12 h	135.81 ± 1.58 ^Bcd^	185.33 ± 5.18 ^Aa^
	24 h	179.35 ± 10.08 ^b^	190.08 ± 13.73 ^a^
	36 h	247.90 ± 15.18 ^Aa^	173.45 ± 19.19 ^Bab^
	72 h	152.40 ± 8.38 ^bc^	165.38 ± 22.46 ^ab^
C1 enzyme (nmol/min/g)	4 h	1113.06 ± 52.41 ^Bc^	2696.35 ± 104.63 ^Ab^
	12 h	3135.52 ± 170.74 ^Aa^	1914.42 ± 204.05 ^Bc^
	24 h	2931.15 ± 131.95 ^ab^	3100.23 ± 88.85 ^b^
	36 h	2693.80 ± 152.47 ^Bb^	3697.37 ± 225.66 ^Aa^
	72 h	2762.01 ± 149.84 ^b^	3058.61 ± 201.28 ^b^
Cx enzyme (nmol/min/g)	4 h	1563.18 ± 105.90 ^d^	1573.46 ± 67.00 ^c^
	12 h	1901.04 ± 89.95 ^Bc^	2396.90 ± 125.45 ^Ab^
	24 h	2081.71 ± 133.63 ^bc^	3131.45 ± 230.17 ^a^
	36 h	2222.04 ± 116.64 ^Bb^	3291.45 ± 103.87 ^Aa^
	72 h	3644.36 ± 133.21 ^Aa^	3001.92 ± 136.63 ^Ba^
NEX enzyme (nmol/min/g)	4 h	213.59 ± 20.57 ^d^	211.70 ± 12.71 ^d^
	12 h	189.97 ± 8.74 ^Bd^	204.64 ± 0.95 ^Ad^
	24 h	321.35 ± 4.32 ^Bb^	438.95 ± 21.32 ^Aa^
	36 h	250.24 ± 5.51 ^Bc^	360.70 ± 27.67 ^Ab^
	72 h	425.62 ± 17.49 ^Aa^	273.22 ± 35.00 ^Bc^

Different lowercase letters in the same column indicate significant differences in data, while different uppercase letters in the same row indicate significant differences. GS, garlic skin; AS, *Artemisia argyi* stalk. BG enzyme, β-glucosidase; C1 enzyme, endo-β-1,4-glucanase; Cx enzyme, exo-β-1,4-glucanase; NEX enzyme, neutral xylanase.

## Data Availability

The data are contained in the article.
